# Navigating Power, Sexual Health, and Communication in Polygynous Marriages: A Qualitative Study Among Hausa Muslim Men in Northern Nigeria

**DOI:** 10.1002/puh2.70157

**Published:** 2025-10-30

**Authors:** Jimoh Amzat, Bello Almu, Ganiyat Amzat, Kehinde Kazeem Kanmodi

**Affiliations:** ^1^ Department of Sociology Usmanu Danfodiyo University Sokoto Nigeria; ^2^ Department of Sociology University of Johannesburg Johannesburg South Africa; ^3^ School of Health and Life Sciences Teesside University Middlesbrough UK; ^4^ Centre for Digital Health Research Innovation and Practice Cephas Health Research Initiative Inc. Ibadan Nigeria; ^5^ Department of Economics Usmanu Danfodiyo University Sokoto Nigeria; ^6^ Office of the Executive Director Cephas Health Research Initiative Inc. Ibadan Nigeria; ^7^ College of Health Sciences Caleb University Imota Nigeria

**Keywords:** Hausa Muslim, male authority, marital communication, Nigeria, polygynous marriage

## Abstract

**Background:**

Polygynous marriage is a socially and religiously recognized marital structure in Northern Nigeria, where cultural and religious beliefs play a central role in shaping family dynamics. This study explores sexual health challenges, power dynamics, and sexual health communication within polygynous marriages among Hausa Muslim men in Northern Nigeria, focusing on the interplay between male authority, cultural norms, and religious beliefs.

**Methods:**

Utilizing a qualitative approach, semi‐structured interviews were conducted with 24 men aged 40 and above, all married to two or more wives. Thematic analysis revealed key insights into how men navigate decision‐making, marital communication, and health management within these culturally complex relationships.

**Results:**

Most participants downplayed the risk of sexually transmitted infections (STIs), with only three explicitly acknowledging a confirmed STI case within the marriage. This low‐risk perception correlates with the near absence of formal healthcare engagement, as most men preferred self‐assessment or traditional remedies. The findings also indicate that male authority is central, with husbands often assuming autonomous control over time‐sharing, financial decisions, and sexual health practices. Although some husbands, though some selectively, consult wives to maintain household harmony, the educated wives are more assertive and subtly influence decisions. Communication about sensitive topics like sexual health is typically indirect, with nonverbal cues favored over explicit discussions, reflecting cultural norms of modesty and privacy. The study also highlights key challenges, such as performance strain, jealousy among co‐wives, and mismatched sexual appetites, which further complicate sexual health decision‐making and emotional well‐being in polygynous households.

**Conclusion:**

Findings contribute to broader understandings of marital dynamics in polygynous contexts, illustrating how men's sexual health and relational strategies are informed by deep‐rooted cultural and religious frameworks. This research provides valuable insight for policymakers and practitioners working to support the unique needs of polygynous families in similar sociocultural settings.

## Graphical abstract

Most participants downplayed the risk of sexually transmitted infections (STIs), with only three explicitly acknowledging a confirmed STI case within the marriage. This low‐risk perception correlates with the near absence of formal healthcare engagement; also, communication about sensitive topics like sexual health is typically indirect.

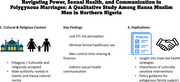

## 1 Introduction

Polygynous marriage, where a man has multiple wives, is a socially and religiously recognized marital structure in many parts of the world, including Northern Nigeria, where cultural and religious beliefs play a central role in shaping family dynamics. Within the Hausa Muslim communities in Nigeria, polygyny is both culturally accepted and religiously sanctioned, aligned with Islamic teachings. In Northern Nigeria, polygyny is deeply embedded in both traditional African and Islamic cultural frameworks. Historically, as in many African societies, it was tied to agricultural economies, where additional wives and children enhanced household productivity and ensured lineage preservation [1]. Among Hausa–Fulani communities, polygyny signified wealth and social standing, with wives contributing to farming, trade, and domestic labor [2]. Islam further institutionalizes the practice, permitting men up to four wives if they could ensure equal treatment (Qur'an 4:3).

In Northern Nigeria, this religious sanction has reinforced polygyny, particularly in rural areas where it serves as a social safety net for widows and unmarried (aged) women [3]. However, urbanization, economic pressures, and Islamic reform movements have led to a gradual decline in its prevalence, especially among educated elites. Despite its cultural and religious roots, polygyny in Northern Nigeria is still significantly prevalent—culturally and religiously entrenched—approximately 30%–50% of ever‐married women in Northern Nigeria are in polygynous unions, with higher rates in rural areas compared to urban centers [4]. Polygyny presents complex family dynamics, including co‐wife rivalries and unequal resource distribution, while still maintaining social and economic functions in many communities.

This unique marital structure introduces complex dynamics that influence power, communication, and health practices within the household [5]. In these settings, husbands are often expected to navigate multiple relationships with a focus on fairness, maintaining harmony among co‐wives, and upholding cultural and religious standards of marital life. However, balancing the needs and expectations of multiple wives presents distinct challenges, especially when it comes to decision‐making authority, managing sexual health, and communicating effectively within the family. Sexual health refers to the physical, emotional, and social well‐being concerning sexual life, encompassing sexually transmitted infection (STI)/HIV risks, reproductive autonomy, relationship dynamics, and access to healthcare. Generally, sexual health is shaped by patriarchal norms, religious values, and gender inequities [6].

The dynamics of power and authority in polygynous marriages often lean toward male‐centered decision‐making, which reinforces the traditional view of the husband as the head of the household [7]; however, wives also have negotiated powers (e.g., using sex and affection) [8]. This study aims to

1. Explore how Hausa Muslim men in polygynous marriages exercise and negotiate authority concerning sexual health;
2. Examine sexual health communication patterns and practices in these unions;
3. Identify factors sexual health and rights (SHRs) challenges and factors, influencing healthcare‐seeking behaviors for sexual and reproductive health (SRH).

Male authority in Hausa Muslim polygynous marriages is maintained through culturally sanctioned decision‐making and indirect communication, which together shape sexual health practices and risk perceptions. Existing literature indicates that although husbands in polygynous marriages wield significant authority, the need for maintaining family cohesion and minimizing conflict leads some men to consult their wives on certain matters, although final decision‐making generally remains with them [9]. The study further investigates how these power dynamics shape the ways in which husbands manage health, particularly sexual health, within a polygynous marriage, where expectations for intimacy and physical health vary across relationships.

The gap in the literature filled by this study lies in its focused, insider‐driven exploration of male perspectives on sexual health, communication, and power dynamics in polygynous marriages within Hausa Muslim communities in Northern Nigeria. Although previous research (e.g., [9, 10]) has addressed gendered inequalities in SRH, most of these studies emphasize women's experiences and vulnerabilities. Few studies provide an in‐depth, culturally grounded exploration of how men in polygynous unions perceive and navigate sexual health responsibilities, marital communication, and male authority—especially from a qualitative and emic (insider) perspective. By focusing on these interconnected areas of sexual health‐related power, communication, and health management, this study provides a deeper understanding of the unique dynamics within polygynous marriages among Hausa Muslim men in Nigeria. Through qualitative analysis, it offers insight into the complex ways men navigate these relationships, balancing individual authority with the cultural and religious expectations that define family life in a polygynous context.

## 2 Methods

This study utilizes a qualitative research design to explore the experiences of Hausa Muslim men in Nigeria who are in polygynous marriages, focusing on themes of power dynamics, communication, and health practices within these relationships. This is phenomenology qualitative method meant to address experiences to understand the essence of a phenomenon as perceived by participants. The methods are particularly suited to capturing the in‐depth, contextualized perspectives of participants, allowing for a nuanced understanding of how cultural and religious norms shape marital practices and decision‐making in polygynous households [11]. The study employs semi‐structured, in‐depth interviews to gather detailed insights into participants’ experiences and perceptions. This approach allows for flexibility in probing relevant topics, ensuring that participants can express personal experiences and cultural values that may not surface in more structured formats.

### 2.1 Study Context and Demographic Patterns of Polygyny

The Northwest geopolitical zone presents a compelling context for studying polygyny, characterized by its predominantly Muslim population and agrarian economy where plural marriages remain culturally embedded. Approximately 38% of married men in this region maintain multiple wives, with prevalence peaking in rural communities (45%) compared to urban centers (25%–30%) [4]. States, like Kano, Kebbi, Sokoto, and Zamfara, exemplify how polygyny intersects with patriarchal norms, religious sanction, and socioeconomic status. Demographic patterns reveal important variations: Although Muslim men exhibit substantially higher prevalence rates (∼35%) than their Christian counterparts (<5%), the practice shows a marked age gradient—peaking among older men (55% at 50+ years) but declining sharply among younger generations (15% under 30) due to economic pressures. Educational attainment and wealth further differentiate these patterns, with uneducated men 2.5 times more likely and wealthier men three times more likely to practice polygyny [4]. Together, these factors demonstrate how traditional, religious, and economic dimensions collectively sustain plural marriage systems in contemporary Northern Nigeria.

### 2.2 Participant Selection

Participants were selected using purposive sampling, targeting Muslim married men in polygynous unions within Hausa communities in Northern Nigeria—specifically from Sokoto, Kebbi, and Zamfara States. Considering that about 38% of men are in polygynous [4], the study participants are many and readily available in the region. Participants were approached through community gatekeepers, including religious leaders and local elders, who helped identify eligible men and facilitated introductions based on trust and cultural rapport. The variables considered in the selection included education (literate and nonliterate), location (rural and urban), and relative economic status. This purposive method was chosen to ensure that participants possess the specific cultural, religious, and marital experiences relevant to the study's focus. Criteria for participation included being a Muslim, male aged 40 or older, currently married to two or more wives, with a minimum of 2 years in polygyny and actively involved in decision‐making within the household. The age requirement ensured that participants had long‐term experience with polygynous marriage dynamics, adding depth to their insights. A total of purposively selected 24 men participated, a sample size considered sufficient for reaching thematic saturation in qualitative studies on focused cultural topics [12].

### 2.3 Data Collection

Data were collected through semi‐structured interviews conducted in Hausa, the participants’ native language, to promote comfortable and open dialogue. Each interview lasted approximately 30–45 min and was audio‐recorded with participant consent. Interview questions were designed to explore three primary areas: sexual health challenges, decision‐making processes, communication strategies, and health practices, particularly in relation to sexual health. Example questions included “How do you make decisions regarding time‐sharing with your wives?” and “Can you describe any health practices you prefer?” This structure allowed for consistent data across interviews while also providing flexibility for participants to elaborate on culturally sensitive issues.

### 2.4 Data Analysis

Although a phenomenological approach guided data collection by focusing on lived experiences, thematic analysis was used to code and interpret recurring patterns within those experiences systematically [13]. The audio‐recorded interviews were transcribed and translated into English by bilingual researchers familiar with Hausa cultural contexts. Analysis involved coding transcripts for recurring themes, such as “sexual health changes,” “sexual health decision‐making,” “nonverbal communication,” and “traditional health practices.” To enhance analytic rigor, two researchers independently coded a subset of the transcripts and compared codes to establish inter‐coder reliability; discrepancies were resolved through discussion and consensus, ensuring consistency and trustworthiness of the thematic framework. NVivo (version 14) was used for the data analysis.

### 2.5 Reflexivity and Positionality

Three of the four authors are Muslims, and the three researcher assistants are male Muslims as well as northerners. The insider status facilitated trust and rapport with participants, yet this shared cultural background risked creating assumptions of “shared” experiences. To mitigate potential bias, we employed bracketing (epoche) techniques throughout data collection and analysis [14]. Bracketing (epoche) in phenomenological research involved consciously setting aside preconceptions, assumptions, and prior knowledge about a phenomenon to authentically understand participants’ lived experiences [15]. Moreover, data interpretations were solely from participants’ raw narratives.

Male research assistants were selected to ensure gender concordance or gender‐matched interview that fosters openness when discussing sensitive marital issues, reducing discomfort and improving data quality in qualitative research [16]. This facilitates the recruitments of the participants. Conscious of power dynamics, the recruitment process emphasized voluntary participation, with interviews conducted in participants’ preferred languages (Hausa) to ensure comfort and authentic expression. We maintained continuous ethical vigilance through reflexive journaling, critically examining how intersecting identities (including gender, education level, and regional affiliation) influenced interactions—particularly when discussing sensitive issue like sexual engagement. This ongoing reflexivity helped balance the advantages of cultural familiarity with necessary methodological rigor.

## 3 Results

Table 1 shows the demographic data of the respondents. The table reveals the study consisted of a group of men predominantly from the Hausa ethnic group in Nigeria, reflecting the cultural context within which these polygynous marriages are practiced. Respondents range in age from 42 to 61, indicating that they are middle‐aged men with significant marital experience. Most have maintained their marriages for a considerable time, with durations between 6 and 30 years, suggesting a long‐term commitment to their polygynous arrangements.

**TABLE 1:** Demographic profile of respondents (*N* = 24).

Variable	Category	Frequency (*n*)
**Ethnicity**	Hausa and Muslims	24
**Age range (**years)	42–49	12
	50–55	8
	56–61	4
**Marriage duration (**years)	6–10	6
	11–20	10
	21–30	8
**Number of wives**	2	16
	3	4
	4	4
**Education level**	Quranic education only	5
	Primary	5
	Secondary	6
	Postsecondary	8
**Occupation**	Farmer and artisan	9
	Civil servant	10
	Businessman	5

Education levels among respondents vary, with most holding primary or secondary school education, and eight having postsecondary education. This range of educational attainment may influence their perspectives on health, communication, and conflict resolution within marriage, as well as the reliance on traditional remedies over formal health services. Occupationally, most respondents are civil servants, with five respondents engaged in business. This employment distribution suggests a degree of stability, which may support the financial demands of maintaining multiple households. The respondents’ occupations and financial status likely impact their ability to meet the needs of multiple wives and children, a factor they often consider in decision‐making.

All participants have more than one wife, typically two, though four respondents reported having four wives. The number of wives correlates with their strategies for time‐sharing and decision‐making, as well as their approaches to managing household dynamics. This demographic analysis provides insight into the social and cultural factors that shape their practices, underscoring the role of age, religion, education, and occupation in their approach to polygynous marriage.

This analysis presents findings from qualitative interviews with men in polygynous marriages. The interviews explore themes of sexual health decision‐making, communication with spouses, time‐sharing, power dynamics, and healthcare access. Notable patterns and participant insights are highlighted, reflecting the complexities and personal experiences of maintaining relationships with multiple wives.

### 3.1 Power Dynamics and Male Authority

In polygynous marriages, the data reflect that power dynamics typically favored male control, especially regarding decision‐making in areas such as time‐sharing, sexual health, and household management. Participants frequently described decision‐making as a role they assumed largely independently, with limited input from their wives (see Matrix 1). As shown in Matrix 1, male‐centric decision‐making was the most frequently reported theme, with men often bypassing wives’ input. This male‐centric approach to power is deeply embedded in cultural and religious norms that position the husband as the primary authority within the marriage. Although some men acknowledged their wives’ opinions, they ultimately made decisions on the basis of their personal preferences or perceived responsibilities, reinforcing the husband's autonomy within these marriages.

**MATRIX 1:** Subthemes on power dynamics and male authority.

Theme	Description	Illustrative quotes with respondent demographics
Male‐centric decision‐making	Men largely control sexual health decisions without input from wives	“I handle everything on my own and keep it to myself without telling them.”—Age 50, 2 wives, primary education, civil servant
Symbolic and indirect communication	Sexual needs and decisions are often conveyed through nonverbal cues, reinforcing male control	“If I bring fish or meat home, they know what's coming.”—Age 58, 3 wives, primary education, civil servant
Permission and compliance expectations	Men expect wives to comply with their sexual needs, equating gifts and care with access to sex	“When you give someone a gift& they naturally want to please you.”—Age 59, 4 wives, postsecondary education, civil servant
Perceived ownership of wives	Some men view their wives as property, reinforcing control over decisions and interactions	“You are the one who married them, and they belong to you.”—Age 42, 2 wives, secondary education, civil servant
Selective favoritism	Favor and time allocation are sometimes based on wives’ sexual performance and behavior	“The one who plays better will get more attention.”—Age 44, 2 wives, postsecondary education, civil servant
Sexual compliance as reward system	Sexual relations and material benefits are tied together in a system of reward and loyalty	“If she gives me joy, I give her more.”—Age 45, 2 wives, primary education, farmer
Minimization of female voice	Women's input in sexual or household decisions is downplayed or avoided to prevent conflict	“If you tell them upfront& they may not like it. But if you keep it quiet, they'll accept it happily.”—Age 50, 2 wives, civil servant
Power justified by capability	Power is justified through male ability to provide, endure, and manage multiple relationships	“I do what I can and leave the rest.”—Age 51, 3 wives, diploma education, civil servant

In some cases, participants did involve their wives in discussions, especially when they anticipated potential conflicts or when establishing routines that might impact household harmony. For example, one man described how he organized a meeting with both wives to discuss the arrangement for child spacing, seeking their input to ensure they understood and accepted the decision. This approach of selective consultation shows that although the final decision rested with the husband, some men acknowledged the importance of minimizing friction by including their wives in certain discussions. However, these consultations were generally described as a means to facilitate compliance, rather than as genuine collaborative decision‐making.

Furthermore, male control extended beyond scheduling and time‐sharing to include other intimate aspects of marital life. When it came to sexual health, for instance, some men noted that they set expectations without necessarily discussing them with their wives. In this dynamic, the husbands’ control over sexual interactions underscored an established power structure where their needs and preferences took precedence, sometimes at the expense of open communication and negotiation with their wives (see Matrix 1). However, this control did not go unchallenged. In some instances, participants reported that certain wives were more vocal or assertive, particularly those with higher levels of education or strong personalities. Some participants shared that their first wife sometimes expressed dissatisfaction with his decisions, especially if she felt unfairly treated or overlooked. Such scenarios suggest that, although husbands generally maintained control, the wives’ responses and levels of assertiveness could subtly influence the balance of power in certain situations. Yet, even in these cases, men retained the final authority, and wives often had to negotiate within the boundaries set by their husbands.

The nature of power dynamics in these marriages is also influenced by the cultural and religious context that reinforces the husband's leadership role. In the interviews, participants emphasized that men are responsible for guiding the family and upholding fairness among wives, which in their view justifies their control over decision‐making. This perspective aligns with religious and cultural teachings that position the husband as the head of the household, entrusted with ensuring the well‐being and order of the family. Many men see this as a duty and a form of stewardship rather than a privilege, feeling that they must balance authority with fairness. For instance, some men explained that they made decisions on the basis of what was deemed best for both wives and is careful to avoid favoritism, underscoring a moral responsibility tied to his role as the decision‐maker.

In essence, the power dynamics in these polygynous marriages reveal a complex interplay between male authority and the subtle influence of wives’ responses. Although men hold the central decision‐making role, they are also mindful of maintaining peace and order within the household, which sometimes necessitates limited consultation with their wives. This arrangement allows husbands to retain control while ensuring that wives feel heard to a degree, which can minimize conflict and foster acceptance of the established structure. However, it also highlights the limitations that wives face in influencing key aspects of their marital life, as their input is largely conditional on the husband's willingness to consider it. Ultimately, the power dynamics observed in these marriages illustrate a balancing act between cultural expectations of male authority and practical considerations of family harmony, underscoring the importance of maintaining a stable, albeit hierarchical, family structure within the context of polygyny.

### 3.2 Perceived SHR Challenges and Decision‐Making

Matrix 2 shows various theme‐concerning perceived risks and SHR challenges in polygynous marriages, indicating several interwoven issues that shape male respondents’ understanding and engagement with sexual health. Most participants downplayed the risk of STIs, with only three explicitly acknowledging a confirmed STI case within the marriage. This low‐risk perception correlates with the near absence of formal healthcare engagement, as most men preferred self‐assessment or traditional remedies. Formal sexual health services were rarely accessed unless a medical emergency arose, and even then, services were often approached with reluctance or secrecy. Respondents described a heavy reliance on traditional knowledge and local concoctions to boost stamina or manage exhaustion, especially in response to performance pressure from multiple wives.

**MATRIX 2:** Subthemes on perceived risks and sexual health and right (SHR) challenges.

Theme	Description	Illustrative quotes with respondent demographics
**Low perception of STI/HIV risk**	Most respondents do not perceive themselves at risk of sexually transmitted infections	“I have never experienced such.”—Age 42, 2 wives, primary education, civil servant “We did HIV tests before marriage and during ANC.”—Age 50, 2 wives, postsecondary education, civil servant
**Minimal use of formal services**	Clinical sexual health services are rarely used. Most rely on self‐assessment or traditional means	“I've never been to the hospital for health services related to sex.”—Age 45, 2 wives, primary education, businessman
**Preference for traditional remedies**	Use of local herbs, roots, and food‐based stimulants as sexual health interventions	“I prepare concoctions with neem, sanga sanga, garlic, and fruits.”—Age 44, 2 wives, Quranic education, business “We inherited herbs from our forefathers.”—Age 45, 2 wives, farmer/artisan
**Exhaustion and performance strain**	Physical exhaustion due to high sexual demand in multiple unions	“There were two instances where I felt helpless& like a mat that can only be folded.”—Age 45, 2 wives, primary education, businessman
**Fear of wife‐to‐wife comparison**	Concerns about favoritism, performance, and competition among co‐wives	“One wife called me *kuti uwar wanka*& that I spoiled the water by sleeping in the other room.”—Age 45, 2 wives, businessman
**Barriers to testing and disclosure**	Fear of suspicion, secrecy, or misunderstanding discourages joint health testing	“If you announce taking them [to the hospital], they'll think something is wrong.”—Age 51, 3 wives, diploma education, civil servant
M**isalignment of sexual desire**	Differences in sexual appetite between spouses cause strain or avoidance	“She is stronger than me in terms of stamina.”—Age 45, 2 wives, farmer/artisan
**Economic strain on sexual expectations**	Meeting sexual and material expectations is financially burdensome	“You can't get what you want unless you provide what she needs—except if you force her.”—Age 56, 4 wives, Quranic education, farmer/artisan

Abbreviation: STIs, sexually transmitted infections.

In addition to physical strain, emotional and relational challenges were evident. Participants often feared comparison and jealousy among co‐wives, leading to secrecy in sexual arrangements and a reluctance to encourage joint health consultations or discussions. The power dynamics in these marriages created a context where unequal sexual appetites led to strain, with some men admitting to physical incapacity to meet the demands of more sexually assertive wives. Financial limitations further compounded this challenge, as respondents indicated that some sexual expectations from wives were tied to material provisions. Altogether, the themes (shown in Matrix 2) expose a confluence of physical, economic, and psychosocial barriers to optimal SHR communication and healthcare‐seeking, shaped by polygyny's unique dynamics.

In the interviews, participants outlined distinct approaches to managing sexual health and time with their wives, reflecting both the complexities and autonomy of these arrangements in polygynous marriages. Most men maintained structured routines for intimacy, which often prioritized their preferences over joint decision‐making, indicating an individualized approach to managing marital expectations. In this context, the scheduling of time with each wife varied, but a common theme emerged: Men frequently decided alone on how to divide nights or days between wives. The consistency of these schedules underscores their desire for control, stability, and order within the household.

One participant (Age 62, 4 wives, primary education, and businessman) described his structured approach as spending two consecutive days with each wife before moving on to the next, describing it as a balanced routine that “helps maintain the relationship and my health” by ensuring that he is not overly taxed by rotating too quickly between wives. Another participant (Age 56, 3 wives, Quranic education, farmer) similarly noted that he preferred 2 days with each wife rather than alternating daily, as this allowed him sufficient rest. These routines were often created by the husbands, and only occasionally discussed with wives after decisions were made, showcasing how male authority was preferred over mutual decision‐making.

Despite the autonomous nature of these decisions, some participants acknowledged that certain wives were more understanding than others. In one case, a husband shared how he structured intimacy by discussing the matter with his wives altogether but faced fewer challenges with one wife than the other. This further highlights the flexibility some men employed to manage the dynamics of multiple relationships while maintaining control. For instance, a participant (Age 51, 3 wives, diploma education, and civil servant) mentioned his personal approach to time management:

I used to spend two days with each wife, starting with the first, then moving to the second and the third& Afterward, I return to the first. I don't make decisions with anyone else; it's my decision.

A few participants also alluded to additional factors influencing their choices in scheduling. For example, one man explained that he delayed intimacy by a few days post‐menstruation as a method of natural birth control, a decision made with his wives’ consent but initiated and directed by him. Another described adjustment made on the basis of each wife's personality and sexual nature, hinting that satisfaction was also part of his consideration in planning schedules.

Thus, although mutual discussions sometimes occurred, these decisions primarily reflected male authority. The husbands' ability to create and control schedules showcases a complex interplay between individual preference, practical considerations, and subtle marital negotiations. These approaches also underscore how autonomy in decision‐making serves as a means to reinforce structure, avoid conflict, and maintain personal health within the unique challenges of a polygynous marriage.

### 3.3 Sharing Time Equitably

Time‐sharing in polygynous marriages emerged as a significant theme, with participants describing various strategies to balance time and attention between multiple wives. Most men adopted structured schedules to ensure fairness and avoid feelings of neglect or jealousy among wives. These schedules varied, with some men adhering to a strict rotation, whereas others allowed flexibility to accommodate personal or family needs, illustrating a balance between equity and adaptability.

For many participants, time‐sharing followed a 2‐day rotation per wife, a pattern that offered stability and ensured that each wife received attention regularly. This structure often minimized potential conflict, as it adhered to Islamic principles of fairness in polygamous arrangements. For instance, one participant noted, “I sit with them, and we decide together how to share time. Some suggest two days per room, others want three.” His approach emphasized the importance of mutual agreement, showing that although men typically made the final decisions, input from wives was occasionally considered to create a harmonious arrangement.

In contrast, some men preferred a more flexible approach, adapting schedules based on situational factors or the temperament of each wife. Flexibility sometimes involved adjusting time to accommodate events, such as travel, illness, or special occasions. For example, one participant explained that when one of his wives traveled, he would stay exclusively with the remaining wife but would then revert to the regular schedule upon the other's return. This approach suggests that, although committed to fairness, men adapted as necessary to maintain overall family balance.

A participant (Age 59, 4 wives, postsecondary education, civil servant) noted:

Yes, I sit with them, and we decide together how to share time. Some suggest two days per room, others want three days& If I have my own room, they come to me when it's their turn.

Another participant, facing a greater number of wives, maintained a personalized rotation system that helped him avoid feelings of physical exhaustion, a practical decision to sustain both his health and his relationship dynamics. He shared that spending 2 days with each wife “helps maintain the relationship and my health” by preventing the fatigue associated with more frequent rotations. This strategy highlights the importance of balancing physical well‐being with time‐sharing obligations, an approach echoed by others who also prioritized personal health while managing multiple relationships.

Although some wives were involved in negotiating schedules, others were simply informed of the arrangement, suggesting that the degree of agency granted to wives varied among participants. However, whether structured or flexible, time‐sharing systems were designed to balance the competing needs of the household, emphasizing equitable attention. Ultimately, these approaches reflected an effort to sustain harmony in the household by minimizing jealousy, managing expectations, and accommodating individual capabilities within the framework of a polygynous marriage.

### 3.4 Communication About Sexual Health

In polygynous marriages, communication about sexual health is complex and often nuanced. As shown in Matrix 3, the findings underscore the limited, individualized, and context‐driven nature of sexual health communication within polygynous households, shaped by gender roles, cultural expectations, and the need to maintain household harmony. The data show that men in these marriages sometimes find it challenging to address sexual health needs directly with their wives. This difficulty may stem from cultural norms around modesty and shyness, or from a sense of maintaining control over the flow of information within the marriage (see Matrix 3). Rather than engaging in open conversations, many participants use indirect methods—signals, gestures, or nonverbal cues—to communicate their sexual desires and needs to their wives.

**MATRIX 3:** Subthemes on communication about sexual health.

Theme	Description	Illustrative quotes with respondent demographics
**Nonverbal/Symbolic communication**	Men often rely on cues like gifts, gestures, or physical touch to express sexual intent	“If I bring home fish or meat, they know it's a sign.”—Age 50, 2 wives, primary education, civil servant
**Individualized conversations**	Communication occurs privately with each wife rather than in group settings	“You can't talk to them about it together because they have different perspectives.”—Age 51, 3 wives, diploma, civil servant
**Avoidance due to jealousy/suspicion**	Group discussions are avoided due to fear of jealousy or misinterpretation	“Discussing with them all together is a calamity.”—Age 51, 3 wives, diploma education, civil servant
**Triggers from wives’ behavior**	Wives sometimes initiate communication through dressing or suggestive gestures	“She might approach me half‐naked& that triggers my desire.”—Age 44, 2 wives, primary education, civil servant
**Cultural constraints and shyness**	Shame or shyness prevents direct communication, especially from wives	“Sometimes they are shy to tell me directly, but I understand their feelings.”—Age 42, 2 wives, Quranic education only, businessman
**Open but selective communication**	Some men report being able to discuss openly, though selectively based on comfort or situation	“Yes, I can talk to them about it.”—Age 45, 2 wives, primary education, businessman
**Role of children or third parties**	Children (especially daughters) sometimes act as informants or mediators in communication	“My daughter Ummi tells me& what her mother said.”—Age 45, 2 wives, primary education, business
**Improvisational communication methods**	Men create relaxed settings (e.g., tea gatherings) to introduce sexual or family issues	“While making tea, I took the opportunity to discuss important matters.”—Age 45, 2 wives, Quranic education only, farming/artisan

Several participants explained that they rarely, if ever, explicitly discuss sexual needs with their wives. Instead, they rely on established routines or subtle cues that the wives have come to recognize over time. For instance, one participant shared that he does not need to verbally request intimacy because his wife understands his intentions based on his actions, such as “bringing home fish or meat,” which she recognizes as a signal that he expects intimacy that evening. This nonverbal approach allows men to convey their desires while avoiding potentially uncomfortable or culturally sensitive conversations about sexual health.

Similarly, other participants noted that their wives also use nonverbal cues to express their readiness or desire for intimacy (see Matrix 3). For example, one man shared that his wife would initiate intimacy by dressing in a certain way or waiting for him after the children had gone to bed, eliminating the need for verbal communication about her intentions. By using these signals, both partners maintain a level of privacy and modesty, which aligns with cultural expectations surrounding discussions of sexuality and reinforces an understanding that each partner should intuitively respond to the other's needs without direct discourse.

Despite the practicality of these indirect methods, they can lead to misunderstandings, as signals and cues are subject to interpretation. For instance, if a husband assumes a particular signal indicates his wife's desire for intimacy, whereas she may have intended it differently, this can cause tension or unmet expectations. However, participants also pointed out that long‐term relationships allow each partner to become attuned to these cues, reducing miscommunication over time. One man noted that “after 20 years of marriage, you just know when your wife needs you, even if she doesn't say it directly.” This shows that although nonverbal communication may initially pose challenges, it can develop into an effective form of communication for some couples.

However, for many, these nonverbal strategies do not completely replace open discussions, especially when it comes to addressing more complex issues of sexual health or concerns. In such cases, men expressed a preference for discussing these topics privately with each wife rather than in a group setting. This individualized approach respects the privacy and uniqueness of each relationship within the marriage. One participant explained that he only discusses sexual health matters with each wife separately, as talking about such matters in front of both wives could lead to jealousy or suspicion. By keeping these conversations private, he believes he can maintain harmony in the household and address each wife's specific needs.

Participants also shared that their discussions about sexual needs often depend on circumstances. Some explained that they only address these topics when necessary, such as when a wife brings up a concern or when there is a significant health issue. One man shared that he sometimes avoids discussing his own health limitations unless they visibly impact his behavior, such as appearing tired or uninterested in intimacy. This approach allows him to manage his own health without confronting sensitive topics unless absolutely necessary, preserving both his privacy and his wives’ expectations.

Overall, communication in these polygynous marriages is characterized by a delicate balance between explicit discussion and implicit signals. Although direct conversations are rare, men have developed methods of conveying their needs in ways that align with cultural expectations and the dynamics of a polygynous household. These practices highlight the importance of understanding and familiarity in long‐term relationships and demonstrate how communication about sensitive topics can adapt to meet cultural norms, even in the absence of open discourse.

### 3.5 Challenges in Accessing Healthcare

In the data provided, most participants indicated a preference for traditional remedies over formal health services when addressing sexual health issues. This inclination likely reflects both cultural norms and practical considerations. Traditional medicine, often passed down through generations, is seen not only as effective but also as culturally appropriate for treating sexual health concerns within these communities. Participants reported various traditional methods, often using locally available herbs, plants, and natural concoctions for stamina and vitality, aligning with the strong reliance on indigenous knowledge and herbal medicine as part of personal and family health management.

Most participants explained that he uses a blend of local herbs for any health issue, including sexual performance. Another shared that he relies on a concoction involving local ingredients specifically known to boost stamina. These remedies are deeply embedded in the local understanding of health and are often seen as effective ways to maintain vitality, especially in a polygynous context where men may feel an added responsibility to meet the needs of multiple wives. Many participants also noted that traditional remedies are readily accessible, affordable, and do not require the time commitment or potential discomfort of formal health consultations, which might be stigmatized in certain communities. One participant (Age 51, 3 wives, diploma education, civil servant) mentioned:

In my 26 years of marriage, I've only experienced one incident where I had a problem and needed an infusion at the chemist. Sometimes, traditional methods work best for me.

In contrast, participants viewed formal health services as necessary only when issues became severe or could not be managed through traditional means. This selective use of healthcare services reflects a common view among participants that formal health interventions should be reserved for emergencies or conditions that traditional methods cannot address.

Additionally, the preference for traditional remedies aligns with a sense of privacy that many participants preferred. Visiting formal health services might require disclosing personal or intimate details that some men were uncomfortable discussing with healthcare providers. This could also be related to limited trust in or access to medical facilities that specialize in sexual health, which might not be widely available or culturally sensitive in certain areas. Participants’ reliance on traditional remedies highlights the role of cultural practices and community knowledge in managing sexual health within these polygynous marriages. This approach offers both practical and symbolic benefits, providing an accessible, private, and culturally resonant method of care that aligns with participants’ health beliefs and their expectations within marital relationships. With this culture of silence and nonverbal communication, discussing sexual infection with the wives presents a challenge.

## 4 Discussion

In polygynous marriages among Hausa Muslim men in Nigeria, findings from qualitative interviews reveal intricate power dynamics, communication strategies, health management practices, and the significant role of cultural and religious contexts. This discussion of findings contextualizes these insights within existing literature, with particular attention to the themes of male authority, indirect communication, health practices, and cultural influences.

### 4.1 Power Dynamics and Male Authority in Decision‐Making

The data reveal that male authority significantly shapes decision‐making within these polygynous marriages, particularly regarding time‐sharing, financial resource distribution, and sexual health. Participants reported an autonomous approach to managing these domains, often deciding independently or with minimal consultation with their wives. This form of decision‐making aligns with findings in other studies on polygynous societies, where husbands are typically regarded as the primary household authorities due to religious, cultural, and social expectations [9, 17]. In Islamic teachings, men are expected to lead and manage their families, and polygyny is permitted under the condition of fairness, which participants referenced as central to their decision‐making.

Other studies also recognized the significant of male authority in their partners’ sexuality [18, 19]. Married women often become victims of sexual health problems due to inability to exercise their SRH rights [18]. Gender‐based inequalities and restrictive gender norms remain significant barriers to women's empowerment and autonomy, particularly in SRH decision‐making. These norms often reinforce power imbalances within relationships, limiting women's ability to make choices about their own bodies [20, 21, 22]. Many women report having little or no control over crucial SRH decisions, such as whether to use contraception or plan the timing and number of pregnancies [19, 23]. Instead, male partners frequently dominate these decisions, reflecting entrenched patriarchal values and societal expectations. This lack of agency can lead to unintended pregnancies, health complications, and reduced access to SRH services, perpetuating cycles of inequality [24]. Additionally, cultural stigmas and inadequate education further marginalize women, depriving them of the knowledge and resources needed to make informed decisions. However, the patriarchal system remains prevalent in most Africa societies; it perpetuates gender inequality and plays a role in putting women's health at risk [10].

However, although husbands generally maintain authority, some degree of consultation with wives was occasionally reported, especially when addressing sensitive issues like child spacing or handling marital conflicts. Studies by Al‐Krenawi [9] suggest that in polygynous marriages, wives often have indirect influence over household decisions by communicating their needs subtly or through negotiation. In this study, husbands occasionally acknowledged their wives’ opinions but ultimately retained control, framing decision‐making as a moral duty tied to ensuring fairness and stability in the household [9, 25]. This dynamic underscores the importance of male authority within cultural expectations of family leadership, emphasizing that although wives may influence certain outcomes, men typically make final decisions. However, women's participation in decision‐making positively influences their SRH [26, 27].

### 4.2 Sexual Health Challenges and Communication Practices

The findings reveal that male participants in polygynous marriages in Northern Nigeria generally perceive low risk regarding sexual health, particularly in relation to STIs. Most respondents reported relying on traditional remedies and self‐assessment rather than accessing formal healthcare services, with only a few acknowledging confirmed STI cases within their households. This preference reflects both cultural familiarity with indigenous healing systems and a broader skepticism with biomedical services—patterns commonly observed in African contexts where traditional medicine is perceived as more accessible, affordable, and culturally congruent [28, 29].

The limited engagement with professional sexual health services reflects deep‐rooted cultural beliefs that prioritize indigenous knowledge and privacy, as well as stigma surrounding open discussions of sexual health [30, 31]. Moreover, performance pressure due to multiple sexual partners contributes to physical exhaustion, further complicating sexual health practices. Emotional stressors such as co‐wife jealousy and unequal sexual appetites also emerged as critical challenges, with men citing fear of comparison and suspicion as reasons for avoiding joint testing or discussions (see also [10]). These dynamics reinforce male autonomy in managing sexual relations, often at the expense of inclusive communication and shared decision‐making. Overall, these findings underscore a complex interplay of cultural norms, masculinity, and health‐seeking behavior that limits optimal SHRs realization.

A recurring theme in the data is the indirect nature of communication within these marriages, particularly around sensitive issues such as sexual health and intimacy. Participants noted that they and their wives relied on gestures, signals, and routines instead of explicit discussions to communicate sexual needs. This aligns with findings from studies by Din et al. [32] and Gourlay [33], which highlight that in societies with conservative values, discussing sexual matters directly is often considered culturally inappropriate. For instance, participants mentioned that specific actions—such as bringing home gifts or changing routines—served as indirect indicators of sexual interest or expectation.

This reliance on nonverbal communication is consistent with norms in many polygynous societies, where modesty and indirect expressions of personal needs are valued [32]. However, it also poses potential risks for miscommunication, especially when husbands or wives misinterpret cues or when cultural expectations discourage open discussions. Although such communication patterns may reinforce privacy and respect between spouses, they can also limit opportunities for addressing and resolving misunderstandings, as noted in research on marital communication in conservative societies [34, 35, 36].

Conservative values, usually based on religious teachings, often fall short in developing comprehensive sexual literacy, as they tend to emphasize moral teachings rather than equipping individuals with practical knowledge about their sexual health [37, 38]. This limited approach may fail to address the complexities of human sexuality, including emotional, psychological, and physical aspects, leaving individuals unprepared to navigate healthy sexual relationships [39]. A key consequence of inadequate sexual education is the lack of open sexual communication between partners, which is critical for understanding each other's sexual needs, wants, and desires. It becomes more complicated with polygynous unions. Conservative values can lead to misunderstandings, dissatisfaction, and unmet needs within relationships, ultimately impacting emotional and relational well‐being. In the Hausa Muslim context, these conservative norms are reinforced through both Islamic teachings and cultural expectations, which may further restrict explicit sexual health discussions between spouses [40]. Moreover, without effective communication skills, individuals in polygynous unions may struggle to negotiate consent or articulate boundaries, increasing vulnerability to exploitation or abuse.

### 4.3 Health Management and Preference for Traditional Remedies

An important aspect of participants’ experiences was their reliance on traditional remedies for managing sexual health rather than formal health services. Most participants described using locally available herbs, as stamina enhancers and preventative health measures. This approach is rooted in cultural traditions that prioritize natural and accessible methods over formal healthcare, a preference also observed in previous studies [30, 31, 41, 42]. In communities where formal health resources are limited or culturally incongruent with community values, traditional remedies are often more accessible, affordable, and viewed as equally effective.

Participants’ limited use of formal health services for sexual health also reflects concerns about privacy and a preference for culturally accepted practices. Research by Gyasi et al. [31] on traditional medicine usage in African contexts highlights that traditional remedies offer privacy, cultural congruence, and convenience, particularly in areas where healthcare systems may not address community‐specific needs effectively. Additionally, relying on traditional remedies over formal healthcare reflects a cultural perspective that views indigenous practices as both familiar and aligned with community health beliefs, as evidenced in studies on indigenous health practices in Africa [31]. The men in this study seem to favor a method that aligns with local norms, where herbal and natural remedies are trusted for their efficacy and cultural relevance.

### 4.4 Influence of Religion and Cultural Norms on Marital Practices

Religion and culture significantly shape practices within these polygynous marriages. Islam, which is the predominant religion among the participants, allows polygyny under certain conditions, such as treating wives fairly and equally. This religious foundation provides a framework within which men exercise authority and strive to maintain fairness in time‐sharing and resource allocation, as also observed in studies by Muradi and Nordin, [43] and Al‐Krenawi [9]. Participants referenced fairness as a core principle guiding their decision‐making, aligning with Islamic teachings that emphasize equal treatment of wives as a condition for polygynous marriages.

Furthermore, cultural norms influence expectations around gender roles, communication, and health practices. In many traditional Hausa communities, male authority and discretion in decision‐making are valued, and women are expected to support and adjust to their husband's leadership [17]. The participants’ descriptions of time‐sharing and financial decision‐making reflect this cultural emphasis on male responsibility and leadership. These norms, deeply rooted in both cultural and religious contexts, reinforce the power structures observed in the study, where men are expected to lead and provide while ensuring family harmony through structured decision‐making and fairness.

### 4.5 Implications for Marital Satisfaction and Household Harmony

The findings suggest that although male authority and structured decision‐making contribute to household stability, they can also create tensions if wives feel neglected or unfairly treated. Research on marital satisfaction in polygynous households shows that fair treatment of wives is essential to maintaining marital harmony, as inequality or favoritism can lead to jealousy and conflict [35, 44, 45]. Although participants in this study generally expressed satisfaction with their arrangements, some acknowledged challenges, particularly with time‐sharing and meeting the diverse needs of multiple wives.

The data also highlight that in cases where men practiced selective consultation, involving wives in decision‐making could foster a sense of inclusion and reduce potential conflicts. This limited form of inclusion, as seen in some participants’ practices, reflects findings by Al‐Krenawi and Graham [46], who suggest that even minimal consultation can improve marital satisfaction and reduce misunderstandings. However, the autonomy retained by husbands shows that the primary authority remains with them, aligning with cultural norms that prioritize male leadership in the household.

## 5 Study Limitations

The study focuses on understanding the dynamics of sexual needs, power relations, and communication within polygynous marriages. This study is one‐sided, interviewing men alone. It emphasizes how men navigate these areas in the context of polygyny, exploring the ways they negotiate their SHRs within a complex web of relationships. However, apart from being one‐sided, it does not explore risk behaviors, treatment‐seeking behaviors, or the specific discussions around risk communication, which are often crucial aspects of sexual health in polygynous contexts.

This research employs a qualitative methodology, which allows for an in‐depth understanding of the role of men within polygynous unions. Through interviews, it captures rich, detailed insights into how these men experience and negotiate SHRs. However, the study's limitations include a small sample size, which may restrict the ability to generalize the findings to the broader population. The qualitative approach, although valuable for understanding personal experiences, may not reflect the full range of the extent or magnitude of certain issues across different regions or communities.

## 6 Conclusion

The findings indicate that most participants downplayed the risk of STIs. This low‐risk perception correlates with the near absence of formal healthcare engagement, as most men preferred self‐assessment or traditional remedies. The findings also underscore the complex interplay between male authority, cultural norms, and marital dynamics in polygynous marriages. Participants’ reliance on traditional health practices, indirect communication, and selective consultation in decision‐making highlight how cultural values and religious beliefs shape marital practices and expectations. These dynamics reflect broader social structures that support male authority while promoting a degree of fairness and stability in polygynous households. The findings suggest potential areas for culturally sensitive interventions, such as communication workshops that respect traditional values while enhancing understanding and open discussion in marital relationships. Additionally, healthcare initiatives that integrate traditional medicine and provide culturally aligned health education may address some of the health challenges reported. Addressing the unique needs of polygynous families, especially in the areas of sexual health risk perception, communication and marital dynamics can provide a foundation for improving family cohesion, marital satisfaction, and overall well‐being in similar contexts.

## Author Contributions

Conceptualisation; data curation; formal analysis; funding acquisition; investigation; methodology; project administration; resources; software; supervision; validation; visualisation; writing original draft; writing review and editing: Jimoh Amzat. Data curation; formal analysis; investigation; methodology; project administration; resources; software; supervision; validation; visualisation; writing–original draft; writing–review and editing: Bello Almu. Conceptualisation; data curation; formal analysis; investigation; methodology; project administration; resources; software; supervision; validation; visualisation; writing–original draft; writing–review and editing: Ganiyat Amzat. Funding acquisition; project administration; resources; writing–review and editing: Kehinde Kazeem Kanmodi.

## Funding

This article is an extract from a main study carried out with funding from Tertiary Education Trust Fund (TETFund) under the Institutional Based Research (IBR) program, 2023. The funder played no influential role in the study design, collection, analysis, and interpretation of data, writing of the report, and the decision to submit the report for publication.

## Ethics Statement

Ethical approval to conduct the study was obtained from Sokoto Health Research Ethics Committee, Sokoto State Ministry of Health, Sokoto State, Nigeria (Ref: SKHREC/110/2024). All participants provided verbal informed consent prior to their participation in the study. All participations were strictly voluntary, and confidentiality was maintained by anonymizing participant identities and storing data securely. Given the sensitive nature of marital and health‐related topics, the study adhered strictly to protocols that safeguarded participants’ privacy and promoted respectful engagement.

## Conflicts of Interest

The authors declare no conflicts of interest.

## Data Availability

The data that support the findings of this study are available from the authors, but restrictions apply to the availability of these data, and they are not publicly available. Data are, however, available from the authors upon reasonable request.
